# Primary Splenic Pregnancy: A Systematic Review of Published Case Reports

**DOI:** 10.3390/jcm15156082

**Published:** 2026-08-05

**Authors:** Goran Augustin, Jure Krstulović, Ante Krešo, Zrinka Hrgović

**Affiliations:** 1 Department of Surgery, University Hospital Centre Zagreb, 10000 Zagreb, Croatia; 2 School of Medicine, University of Split, 21000 Split, Croatia; jkrstulovic@kbsplit.hr (J.K.); zrinka.hrgovic@mefst.hr (Z.H.); 3 Department of Surgery, University Hospital of Split, 21000 Split, Croatia; 4 Department of Ophthalmology, University Hospital of Split, 21000 Split, Croatia; 5 Department of Family Medicine, Split-Dalmatia Health Center, 21000 Split, Croatia

**Keywords:** splenic pregnancy, diagnosis, Augustin’s triad, treatment, outcome, systematic review

## Abstract

**Background/Objectives**: Primary splenic pregnancy (PSP) is a rare and life-threatening form of extrauterine pregnancy. Evidence is limited to case reports, and detailed analyses of all aspects of PSP are lacking. The objective is to review and summarize the clinical features, diagnostic methods, management strategies, and maternal outcomes of PSP. **Methods**: Review followed PRISMA 2020 guidelines. We conducted a literature search to identify all published reports of PSP in PubMed, PubMed Central, and Google Scholar published until September 2025. The search items included: ‘splenic pregnancy’, ‘splenic ectopic pregnancy’, ‘ectopic pregnancy’, ‘pregnancy’, and ‘spleen’. Additional studies were identified by reviewing the reference lists of retrieved studies. We included all available full-text cases, with the inclusion criterion being PSP and the exclusion criterion being secondary splenic pregnancy. The data on clinical presentation, diagnostics, management, and outcomes were extracted and summarized descriptively. **Results**: Sixty published cases of PSP were collected. The average maternal age was 30.3 years, and most cases were reported in Asia. No consistent pattern of potential risk factors was observed; cases occurred between 4 and 14 weeks of gestation. Abdominal pain was the most common symptom (86.7%), often localized to the left upper quadrant. Hypotension and hemoperitoneum were present in 41.7% and 60.0% of cases, respectively. Ultrasound was the most used initial imaging modality, whereas CT and MRI showed high concordance with the final diagnosis. Most implantations occurred on the inferior and superior poles of the spleen. Surgical intervention was the predominant approach (90%), primarily involving laparotomy with splenectomy or partial splenectomy. Antibiotic use was rarely documented. Medical management was reported in selected cases, but no βhCG threshold for PSP can be inferred. Maternal survival was almost universal; the single reported death occurred in the earliest case. **Conclusions**: PSP should be excluded when left-sided abdominal pain, particularly with intra-abdominal bleeding, accompanies an empty uterus and elevated βhCG. No reliable βhCG threshold for treatment selection has been established, and management should be individualized based on clinical condition, diagnostic imaging, and available expertise, based on low level of evidence. Although maternal survival is excellent, spleen-preserving and non-surgical approaches remain rare.

## 1. Introduction

Primary splenic pregnancy (PSP) is an exceptionally rare, life-threatening form of extratubal ectopic pregnancy in which a fertilized ovum implants on the splenic surface. Because the spleen is a highly vascular and fragile organ, it cannot support placental growth, making the condition highly susceptible to rupture and massive intra-abdominal bleeding during the first trimester. Clinical presentation differs from that of other intra-abdominal conditions, but clinicians rarely suspect it. Consequently, diagnosis and treatment are delayed. It is important to distinguish PSP from secondary abdominal pregnancy. Secondary abdominal pregnancy results from an initial tubal pregnancy that is expelled (tubal abortion) or ruptures, after which the embryo and trophoblastic tissue remain viable, reimplant within the peritoneal cavity, establish a new blood supply, and continue to develop. In contrast, PSP represents implantation on the spleen without prior tubal implantation. Another uncommon ‘’mirror’’ form on the opposite side of the abdomen is primary hepatic pregnancy [1], also extremely rare. Due to their extreme rarity, the natural history, diagnostic processes, and effectiveness of various management options for both conditions are not well established. Furthermore, the pathophysiological mechanisms underlying implantation of the gestational sac within splenic tissue remain poorly understood, and current explanations largely rely on theoretical assumptions drawn from individual case reports. Given the small number of published cases and only one review, which was not a systematic review and lacked formal analysis and conclusions [2], we conducted a systematic review of 60 published cases of PSP, beginning with the first documented case in 1936 [3]. Given the exceptional rarity of PSP, higher levels of clinical evidence, such as cohort studies or randomized trials, are currently unavailable. Therefore, published case reports and small case series represent the only available evidence for summarizing the clinical presentation, diagnostic approaches, management strategies, and maternal outcomes of this condition. This systematic review aimed to identify and summarize all published cases of PSP and to describe their clinical presentation, diagnostic methods, management strategies, and maternal outcomes.

## 2. Materials and Methods

**Figure 1:**
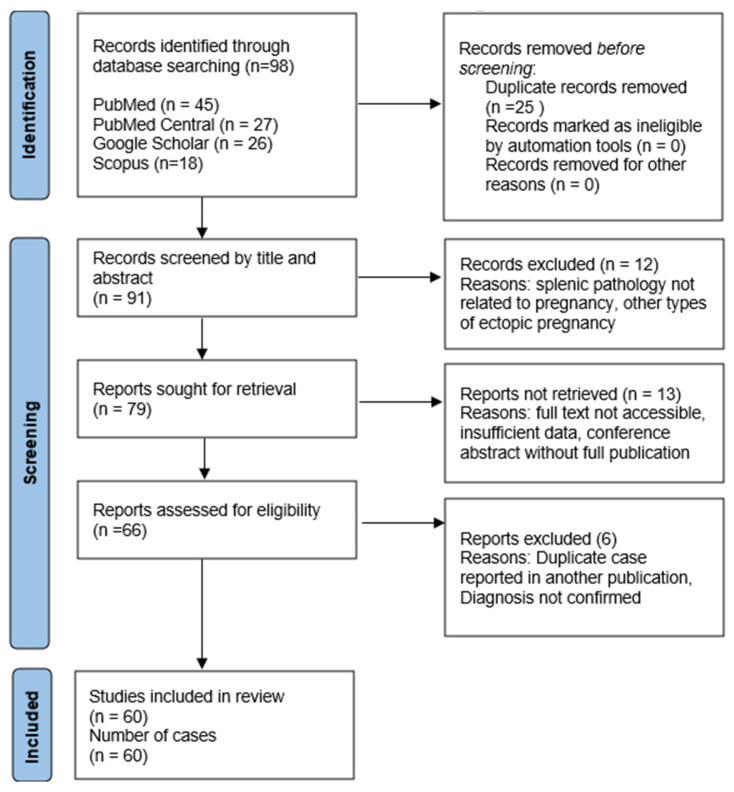
PRISMA flowchart for primary splenic pregnancy.

Two authors (A.K. and Z.H.) independently conducted the literature search in PubMed, PubMed Central, Google Scholar, and Scopus. PubMed was selected as the primary biomedical database, while PubMed Central, Google Scholar, and Scopus were additionally searched to maximize retrieval of freely available full-text articles, older publications, and rare case reports that may not be comprehensively indexed in traditional biomedical databases. To further ensure comprehensive literature retrieval, an additional search of the Scopus database was performed using the same search strategy and eligibility criteria. No additional eligible cases were identified. The collected data were analyzed in accordance with PRISMA 2020 guidelines [4] (Figure 1). PRISMA Checklist 2020 is in the Supplementary Material. The study was not registered. The literature search was last conducted on 30 September 2025 and included all publications available up to that date. No language restrictions were applied. The search strategy included combinations of the terms ‘splenic pregnancy’, ‘splenic ectopic pregnancy’, ‘ectopic pregnancy’, ‘pregnancy’, and ‘spleen’. Additional studies were identified by reviewing the reference lists of the retrieved studies. The complete search strategy is provided in Appendix A. We included all available full-text cases and case series. Conference abstracts without a subsequently published full-text article, reports with unavailable full text, and reports containing insufficient information to confirm the diagnosis of PSP were excluded. The inclusion criterion was PSP; secondary abdominal (ectopic) pregnancies were excluded per the predefined eligibility criteria. The diagnosis of PSP was accepted as reported by the original authors and supported by operative findings, histopathological confirmation, or imaging findings when available. There were no cases of heterotopic pregnancy with splenic pregnancy; therefore, we did not discuss this condition in the inclusion/exclusion criteria.

This study is exempt from ethics approval as we collected data from published cases.

This review was not prospectively registered because the protocol was not developed before the literature search began. The lack of prospective registration is acknowledged as a limitation of the study. In addition, a formal methodological quality or risk-of-bias assessment was not conducted because the review consisted exclusively of case reports and small case series, and the primary aim was descriptive synthesis rather than comparative evaluation. Given the review’s descriptive nature and the rarity of PSP, we considered that formal appraisal would have had limited influence on the interpretation of the findings.

### 2.1. Study Selection

Two authors (A.K. and Z.H.) independently screened the titles and abstracts of all retrieved records. Full-text articles deemed potentially eligible were then assessed for inclusion. Disagreements regarding study eligibility were resolved through discussion and consensus among the investigators. Additional eligible studies were identified through manual screening of reference lists. Duplicate records were removed during screening. When multiple publications reported the same case, only the most comprehensive report was included in the final analysis.

### 2.2. Data Extraction

Two authors (A.K. and Z.H.) independently extracted data from the included articles using a predefined data extraction form based on standardized data collection criteria. Although the form was not formally pilot-tested, the use of predefined extraction criteria, independent data extraction by two reviewers, and resolution of discrepancies through discussion and consensus were intended to maximize the reliability and consistency of the collected data. Data were summarized using descriptive statistics. Continuous variables are presented as medians with ranges or means with standard deviations, as appropriate, whereas categorical variables are presented as frequencies and percentages.

Demographic/clinical data included maternal age, comorbidities, prior abdominal surgeries, and continent/region. Obstetric data included gestational age, parity, number of prior deliveries/Cesarean sections (CS), prior spontaneous miscarriages/pregnancy losses, prior ectopic pregnancies, infertility/ovulation induction, intrauterine device (IUD) use, and oral contraceptive use. Clinical presentation parameters included pain characteristics (duration, progression, location), gynecologic symptoms, and other symptoms. Physical examination included tachycardia, hypotension, fever, Kehr’s sign, abdominal tenderness, guarding, rigidity, distension, bowel sounds, vaginal examination, and the cervical excitation test. Diagnostics included a urine pregnancy test, serum βhCG levels, transabdominal and transvaginal ultrasound (US), abdominal CT, chest CT, abdominal MRI, chest X-Ray, and abdominal X-Ray. Management parameters included medical, radiologic, or surgical treatment, type of abdominal wall access, and type of surgical procedure in operated patients. Other parameters collected included differential diagnosis, location of the gestational sac on the spleen, maternal outcome, and postoperative complications.

Missing data were not imputed. Analyses were conducted using the available data for each variable, and denominators were reported where appropriate. The proportion of available observations therefore varied across variables due to inconsistent reporting in the published case reports. Variable-specific denominators are reported throughout the Results to ensure transparency regarding data availability.

### 2.3. Statistical Analysis

All statistical analyses were conducted in JASP (open source, Amsterdam, The Netherlands, version 0.18.3) using the JASP Team (2024). We calculated means and SDs, medians, and interquartile ranges for continuous variables. For categorical variables, we calculated frequencies and proportions. A *t*-test was applied to continuous variables, while χ^2^ tests or Fisher’s exact tests were used for categorical variables. Given the descriptive nature of the included case reports and the absence of a predefined comparison cohort, all statistical comparisons should be considered exploratory and interpreted with caution. No causal or inferential conclusions can be drawn from these analyses.

## 3. Results

Of the 98 cases identified in our initial search (Figure 1), 60 met the criteria for PSP. The characteristics of the included case reports, including demographic, clinical, diagnostic, management, and outcome data, are summarized in Supplementary Table S1.

### 3.1. Risk Factors

The mean maternal age was 30.3 years, with a wide range (17–42). Geographical distribution, in descending order, was Asia 34 (56.7%), Europe 13 (21.7%), North America 7 (11.7%), South America 2 (3.3%), and Oceania 4 (6.7%), with no published cases in Africa. The obstetric history of patients with PSP is presented in Table 1. Based on the descriptive data in Table 1, no consistent pattern of potential risk factors emerges; however, the absence of a control group precludes drawing conclusions about risk factors. An IUD was reported in 15%, and a previous ectopic pregnancy in 3.3% of cases. However, these findings cannot be interpreted without comparison to an appropriate control population.

### 3.2. Symptomatology and Clinical Examination

The most common set of PSP symptoms constitutes ***Augustin’s triad***, which includes:

- Spontaneous initial left upper quadrant (sometimes flank/lumbar) pain,
- Amenorrhea of approximately 2 months,
- Vaginal bleeding/discharge.

This triad occurs in more than half of patients (Table 2). Nausea or vomiting was present in 15%, while other symptoms were infrequently reported. Depending on the duration and amount of bleeding, the pain intensifies and localizes to the left side of the abdomen or becomes generalized. No apparent difference in symptom duration was observed between ruptured and non-ruptured cases; however, interpretation is limited by the small number of reported cases and incomplete reporting.

Abdominal tenderness and tachycardia are common. The rate of hypotension was similar in ruptured and non-ruptured cases; however, no meaningful comparison can be made given the limited sample size. *Kehr’s sign* is the occurrence of acute pain in the tip of the shoulder (classically the left shoulder) caused by irritation of the diaphragm. It is a referred pain and is widely used as an indicator of internal bleeding or inflammation. It was described in 20% of patients. The rate of vaginal examinations (Table 3) was only 40%, indicating that pregnancy often goes unrecognized despite amenorrhea. A urine pregnancy test was performed in just half of the patients. The median gestational age at diagnosis was 7 weeks (IQR 6–9; range 4–14 weeks; *n* = 43). Cervical examinations were seldom performed.

### 3.3. Diagnosis

**Figure 2:**
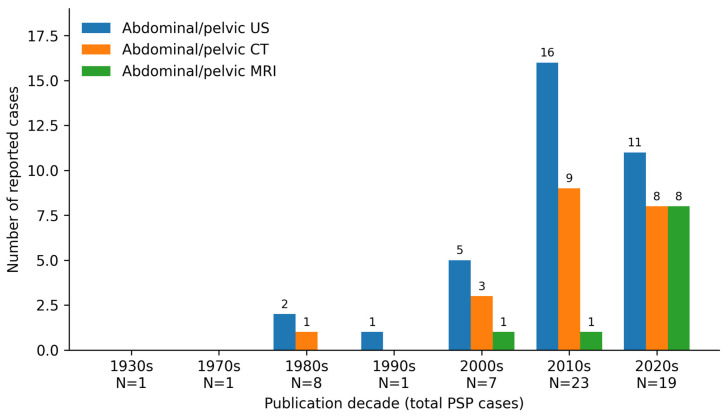
Absolute counts of reported imaging modalities by decade of publication. The total number of published PSP cases for each decade is shown below its label. Bars show absolute counts rather than percentages to avoid overinterpretation of early decades with very small denominators. US: abdominal/pelvic ultrasound; CT: computed tomography; MRI: magnetic resonance imaging; PSP: primary splenic pregnancy.

Transvaginal and transabdominal ultrasound were performed in nearly half of the patients (Table 3). Abdominal CT was performed in one-third of the patients. Other imaging modalities (Table 3) were rarely used, and no patients underwent a plain abdominal X-Ray. The preoperative differential diagnosis was mentioned in 70% of cases (Table 3). Figure 2 presents the absolute numbers of abdominal/pelvic US, CT, and MRI use by publication decade; the total number of reported cases in each decade is shown on the *x*-axis because percentage-based trends are unstable in decades with very small denominators, particularly the 1990s (*n* = 1).

Although transabdominal US has relatively high accuracy (71.4%), abdominal CT and MRI are highly accurate and diagnostic (95.2% and 100%, respectively).

### 3.4. Treatment

Almost a third of patients initially presented to an ambulatory/outpatient clinic (Table 4 and Table 5). Laparoscopic treatment was completed in 21.7%, and conversion to laparotomy occurred in 10%. In the rupture-status comparison, the operative approach (no operation, laparoscopy, or laparotomy, including conversions) did not differ significantly between ruptured and non-ruptured pregnancies (*p* = 0.75; Table 6). The median gestational age at diagnosis appeared higher in the spleen-preserving group (10 vs. 6 weeks; Table 7); however, meaningful comparison is limited by the extremely small number of spleen-preserving cases (*n* = 2). Therefore, no firm conclusions can be drawn.

The frequency of hemoperitoneum was similar between the splenectomy and spleen-preserving groups (64.8% vs. 50.0%; *p* = 1.00; Table 7). The presence of a splenic cyst, mass, or tumor also did not differ between groups (*p* = 1.00; Table 7). Lesion size could not be compared because the database lacked a continuous size variable. No clear pattern of splenic implantation site was observed across the reported cases, although most were implanted on either the superior or inferior pole of the spleen (Table 4).

**Table 4:** Treatment and operative characteristics in women with primary splenic pregnancy (*n* = 60).

Characteristic	Category	Value, n (%)
Presentation setting		
The first presentation	Emergency department	41 (68.3%)
	Outpatient/ambulatory clinic	19 (31.7%)
Overall surgical management		
Surgical exploration/operation *	No surgery/no operative exploration	3 (5.0%)
	Diagnostic exploration only (no splenic intervention)	1 (1.7%)
	Exploratory laparotomy/laparoscopy with splenic operation	56 (93.3%)
Abdominal surgical approach	No operation	3 (5.0%)
	Laparoscopy	14 (23.3%)
	Laparotomy	37 (61.7%)
	Laparoscopy converted to laparotomy	6 (10.0%)
Splenic procedures **†**		
Splenic procedure performed	No splenic operation	4 (6.7%)
	Splenectomy/partial splenic resection	54 (90.0%)
	Spleen-preserving removal of gestational tissue only	2 (3.3%)
Non-surgical/adjuvant treatments **‡**		
Systemic methotrexate (i.m.)	Any use	4 (6.7%)
	Used without splenic operation	3 (5.0%)
	Combined with surgery	1 (1.7%)
Methotrexate injection into the gestational sac	Any use	2 (3.3%)
	Used without splenic surgery	1 (1.7%)
	Combined with surgery	1 (1.7%)
Invasive radiological treatment		4 (6.7%)
–splenic artery embolization		3 (5.0%)
–CT/US-guided intralesional MTX + KCl		1 (1.7%)
Invasive radiological treatment without splenic surgery	Cases with radiological treatment and no splenic operation	3 (5.0%)
Invasive radiological treatment combined with surgery	Cases with radiological treatment and laparoscopic/laparotomic approach	1 (1.7%)
Any pharmacologic or radiological therapy (systemic MTX, local MTX, and/or radiological treatment)	Any of the above	6 (10.0%)
	Used without splenic operation	3 (5.0%)
	Combined with surgery	3 (5.0%)
Intraoperative findings related to the spleen		
Hemoperitoneum	Present	36 (60.0%)
	Absent/not reported	24 (40.0%)
Bleeding spleen/ruptured capsule/laceration	Present	27 (45.0%)
	Not described/absent	33 (55.0%)
Ectopic pregnancy tissue visible on the spleen §	Fetus/gestational sac/trophoblastic tissue described on the spleen	20 (33.3%)
	Not described/absent	38 (63.3%)
	Unknown (missing)	2 (3.3%)
Cyst/mass/tumor on spleen	Described	20 (33.3%)
	Not described/absent	40 (66.7%)
Site of splenic ectopic implantation **║**		
Site of implantation on the spleen	Unknown	18 (30.0%)
	Superior pole	13 (21.7%)
	Inferior pole	15 (25.0%)
	Splenic hilum	7 (11.7%)
	Dorsal pole + Lateral convex surface	5 (8.4%)
	Other/unspecified location	2 (3.3%)

* “No surgery/operation/diagnostic exploration/exploration with operation” is based on the variable *No surgery/operation* (0)/*Only diagnostic exploration without other operation* (1)/*Exploration with operation* (2) from your dataset. † The original variables for operating/surgical procedure codes were: 0 = no operation; codes 1–4 = splenic operations (various forms of splenectomy or splenic resection); 5 = spleen-preserving removal of fetus/gestational tissue. “No splenic operation” includes the 3 cases without operative exploration and the 1 case with diagnostic exploration only. The 56 patients with a splenic operation include 54 with splenectomy/partial splenic resection and 2 with spleen-preserving removal of gestational tissue. ‡ Non-surgical treatments (systemic MTX, local MTX, and invasive radiological procedures) are reported per patient. “Used without splenic operation” denotes treatment in patients without splenectomy, partial splenic resection, or spleen-preserving removal of gestational tissue; “combined with surgery” denotes concurrent laparoscopic or open splenic surgery. Categories are not mutually exclusive across treatment modalities (e.g., some patients received both systemic MTX and splenic artery embolization). The six-patient comparator group in Table 8 is defined by splenic resection status and should not be interpreted as the systemic MTX cohort. § In the variable *Ectopic pregnancy/Pregnancy formation/Fetus/Placenta/Gestational sac/Trophoblast tissue on spleen*, code 0 combines “absent” and “not available”; therefore, only code 1 (description present) can be interpreted as a definite visualization of ectopic tissue on the spleen. ║ The coding dictionary for the implantation site is partially truncated in the column header. Codes explicitly visible are: 0 = unknown, 2 = superior pole, 4 = splenic hilum, 6 = dorsal pole, 7 = lateral convex surface. Code 3 corresponds to “inferior pole” (partially visible as “…pole”), while code 1 was grouped as “other/unspecified location”.

In most cases (90%), splenectomy or partial splenectomy was performed. Spleen-preserving removal of gestational tissue was performed in 3.3% of cases presenting with minimal bleeding. Antibiotic administration was rarely documented in the published reports; when it was, it was explicitly noted as not given (Table 5).

**Table 5:** Maternal outcomes and histopathological findings in women with primary splenic pregnancy (*n* = 60).

Characteristic	Category	Value, n (%)
Maternal outcome		
**Maternal survival**	Alive	59 (98.3%)
	Dead	1 (1.7%)
Postoperative course		
**Overall postoperative course**	Uneventful recovery	54 (90.0%)
	Postoperative ileus	1 (1.7%)
	Death	1 (1.7%)
	Unknown/not reported	4 (6.7%)
**Other specific postoperative complications**	Intra-abdominal abscess/collection, wound infection, thromboembolism, respiratory complications, or need for surgical re-intervention	Not systematically reported; not quantifiable across cases.
Histopathology		
**Histopathological examination of the spleen**	Performed	50 (83.3%)
	Not performed (coded as 0)	10 (16.7%)
Fetal tissue/gestational sac on histology *	Present among documented histology	3/50 (6.0%)
	Not identified in documented histology	47/50 (94.0%)
Trophoblastic tissue/chorionic villi *	Present among documented histology	46/50 (92.0%)
	Not identified or not specified in documented histology	4/50 (8.0%)
Antibiotic therapy		
**Prophylactic antibiotics**	Documented as given	0 (0.0%)
	Not documented as given	60 (100.0%)
**Therapeutic (postoperative) antibiotics**	Documented as given	2 (3.3%)
	Not documented as given	58 (96.7%)

* Histology subanalyses are presented for the 50 patients with documented histopathological examination. The original binary fields merged “absent” with “histology not done”; therefore, the 10 patients without documented histology were excluded from the denominator-specific histology rows. Antibiotic rows indicate documentation of antibiotic use in the published reports; the absence of documentation was not interpreted as confirmation of non-administration. βhCG normalization: Post-intervention serum βhCG values were available for only 13 women through postoperative day 7; in all recorded measurements, levels remained above the non-pregnant range. Therefore, the time to achieve βhCG negativity could not be reliably summarized from the available data.

Non-surgical or adjuvant treatments include systemic (intramuscular) methotrexate (MTX) or MTX injection into the gestational sac. Systemic MTX was rarely combined with surgery. Given the small number of cases, it is difficult to determine whether intragestational MTX injection should be combined with surgery. Recently, radiological treatment (splenic artery embolization and/or CT- or US-guided intralesional MTX + KCl) has been used more frequently in hemodynamically stable patients. These treatment categories overlap and should not be considered mutually exclusive.

To avoid conflating systemic MTX use with treatment-group denominators, Table 8 compares patients who underwent splenectomy or partial splenectomy (*n* = 54) with those managed without splenic resection, including no splenic operation, diagnostic exploration only, or spleen-preserving removal (*n* = 6). Pre-intervention serum βhCG was available for 42 patients (37 vs. 5), first post-intervention βhCG for 13 patients (9 vs. 4), and paired early relative βhCG decline for 12 patients (8 vs. 4). These data are descriptive only because the comparison is severely underpowered.

**Table 6:** Comparison between ruptured and non-ruptured primary splenic pregnancy.

Variable	Ruptured Splenic Pregnancy (n = 24) †	Non-Ruptured Splenic Pregnancy (n = 36)	p-Value *
**Duration of symptoms, days**	1.5 (IQR 0–7.8; range 0–31; *n* = 22)	1.0 (IQR 0–7.0; range 0–60; *n* = 35)	0.95
**Hypotension (shock surrogate)**	10/24 (41.7%)	15/36 (41.7%)	1.00
**Hemoperitoneum**	12/24 (50.0%)	24/36 (66.7%)	0.28
**Abdominal approach**			0.75
–No operation	1/24 (4.2%)	3/36 (8.3%)	
–Laparoscopy	6/24 (25.0%)	7/36 (19.4%)	
–Laparotomy (including conversion)	17/24 (70.8%)	26/36 (72.2%)	

† Ruptured pregnancy was defined as an explicit splenic rupture, active splenic bleeding, splenic laceration, or a ruptured splenic lesion as described in the original report; hemoperitoneum alone was not used to define rupture because it was also reported in cases without explicit rupture. * Duration of symptoms: Mann–Whitney U test; hypotension and hemoperitoneum: Fisher’s exact test; abdominal approach (3 × 2 table): χ^2^ test.

**Table 7:** Comparison of splenectomy and spleen-preserving management in primary splenic pregnancy.

Variable	Splenectomy (n = 54)	Spleen-Preserving Removal of Gestational Tissue (n = 2)	p-Value
**Gestational age at diagnosis, weeks**	6 (IQR 6–9; range 4–14; *n* = 39)	10 (IQR 9–11; range 8–12; *n* = 2)	0.18 ^a^
**Hemoperitoneum**	35/54 (64.8%)	1/2 (50.0%)	1.00 ^b^
**Cyst/mass/tumor on spleen**	19/54 (35.2%)	1/2 (50.0%)	1.00 ^b^

^a^ Mann–Whitney U test (two-sided) for gestational age. ^b^ Fisher’s exact test for 2 × 2 tables (presence vs. absence of hemoperitoneum or cyst/mass/tumor).

**Table 8:** Outcomes and βhCG dynamics according to splenic resection status.

Variable	Splenectomy/Partial Splenectomy (n = 54)	No Splenic Resection/Spleen-Preserving Management (n = 6)	p-Value *
Maternal outcome and complications			
Maternal death, *n* (%)	1 (1.9%)	0 (0.0%)	1.00
Any postoperative complication or death, *n* (%)	2 (3.7%)	0 (0.0%)	1.00
Serum βhCG dynamics			
Pre-intervention serum βhCG, mIU/mL	21,931 (9278–34,279; 46–711,431); *n* = 37	8399 (8150–38,913; 3400–53,000); *n* = 5	0.73
First post-intervention serum βhCG, mIU/mL	2986 (2000–5390; 657–21,472); *n* = 9	2556 (1796–6448; 552–17,092); *n* = 4	0.71
Relative decrease in βhCG from pre- to first post-intervention, %	73.4% (59.1–80.1); *n* = 8	64.5% (45.7–78.0); *n* = 4	0.68

* Continuous variables: Mann–Whitney U test (two-sided). Categorical variables: Fisher’s exact test (two-sided). The no splenic resection/spleen-preserving group includes four patients without splenic resection and two patients with spleen-preserving removal of gestational tissue; this group is not equivalent to the systemic MTX subgroup. All comparisons are exploratory and should be interpreted descriptively.

### 3.5. Maternal and Fetal Outcome

Maternal survival was 98.3% (59/60), with a single maternal death reported in the earliest case from 1936 [3]. In the resection-status comparison, maternal death (1/54 vs. 0/6; *p* = 1.00) and the composite endpoint of postoperative complication or death (2/54 vs. 0/6; *p* = 1.00) were too sparse to allow meaningful inference (Table 8). Symptoms, including abdominal pain, typically precede significant bleeding, enabling early and effective intervention in otherwise young and healthy women. An uneventful recovery was reported in 90% of patients (Table 5). Among women with documented histopathology (*n* = 50), trophoblastic tissue and/or chorionic villi were identified in 46/50 (92.0%), and fetal tissue/gestational sac was explicitly recorded in 3/50 (6.0%).

No fetus has reached viability.

## 4. Discussion

PSP is an exceedingly rare condition. A recent review [2] reported 43 cases, but 44 were actually included because one was duplicated—initially published as an abstract/poster in one journal [5], and later as a full article in another [6]. We compiled and systematically reviewed 60 documented cases over the past 90 years, while more than 10 articles were unavailable. Given the small number of cases, conclusions should be drawn cautiously. Interestingly, from Werner Nagel’s first description in 1936 [3], it took 41 years for a second case to be documented by Mankodi et al. [7] in 1977.

Based on the included case reports, the exact mechanism by which a fertilized ovum reaches the spleen remains unknown. Although several hypotheses have been proposed, none has been experimentally confirmed. Most implantations occur at the inferior and superior poles of the spleen. The data did not show a consistent pattern of potential risk factors [8]. Interpretation is limited by incomplete reporting and the lack of a control group. Although 15% of women were using an IUD, the association remains unclear. IUD use does not increase the overall risk of ectopic pregnancy [9]; however, ectopic pregnancies are more common with an IUD in place [10]. Furthermore, IUD use is a potential risk factor for non-tubal ectopic pregnancies, particularly ovarian ectopic pregnancy [11].

When ***Augustin’s triad*** is considered, the clinical presentation is straightforward. To our knowledge, this triad has not been previously described in association with other conditions. Interestingly, *Kehr’s sign*, a referred shoulder tip pain from diaphragmatic irritation, is observed in only 20% of PSP patients. One would expect a higher frequency given the left upper intraperitoneal bleeding from splenic bleeding near the left diaphragm. A presentation similar to PSP but without vaginal bleeding/discharge occurs in pregnancies with spontaneous rupture of a renal artery aneurysm [12], but these are normal intrauterine pregnancies. Differences include sharp, excruciating lumbar pain initially and hypotension, often with collapse in later stages [8]. In PSP, initial pain is not very intense, and nearly a third of patients first seek outpatient care. With prolonged bleeding, the pain spreads and eventually becomes generalized. Interestingly, hypotension occurs equally often in ruptured and non-ruptured PSP cases. An explanation could be minimal splenic bleeding and physiological, harmless hypotension during early pregnancy, with values of about 5–10 mmHg (systolic/diastolic) in the first 20–24 weeks. It is caused by decreased vascular resistance, increased blood volume, and hormonal changes, often leading to dizziness or fatigue. If more severe hypotension accompanies abdominal pain, the condition should be taken seriously. However, given the limited number of cases and incomplete reporting, this observation should be interpreted cautiously.

Although transabdominal US has relatively high accuracy (71.4%), abdominal CT and MRI are highly accurate and diagnostic (95.2% and 100%, respectively). MRI has been commonly used since 2010.

Splenectomy or partial splenectomy was the most common procedure, while spleen-preserving removal of gestational tissue was performed in 3.3% of cases, with increasing use of laparoscopy [13]. When technically feasible and clinically appropriate, spleen-preserving surgery should be considered. Antibiotic use was rarely documented, but the lack of reporting should not be interpreted as confirmed non-administration. Non-surgical or adjuvant treatments include systemic (intramuscular) MTX or intra-gestational MTX injections, as well as image-guided approaches such as splenic artery embolization and/or CT/US-guided intralesional MTX + KCl in hemodynamically stable patients [14]. Because treatment categories overlap and the no-resection/spleen-preserving group contained only six patients, no between-group inference can be drawn regarding complications or βhCG decline. Percutaneous embolization alone, with postoperative βhCG monitoring, is sometimes sufficient when βhCG normalization is confirmed [15].

In tubal ectopic pregnancy, serum βhCG is commonly considered alongside clinical and US findings when selecting candidates for medical management, although treatment decisions are not based on βhCG levels alone [9,16]. However, these treatment algorithms cannot be directly extrapolated to PSP because of its distinct anatomical location, different bleeding risk, and the absence of comparative studies. In the present review, management without splenectomy or partial splenectomy was reported in only six patients, and pre-intervention βhCG values in this group were available for only five patients (range 3400–53,000 mIU/mL), precluding identification of any clinically meaningful threshold. Furthermore, published case series of non-tubal ectopic pregnancies at different anatomical locations have not demonstrated a consistent association between serum βhCG levels and treatment success [17]. Therefore, based on the currently available evidence, no evidence-based βhCG threshold can be recommended for PSP, and treatment decisions should be individualized according to the patient’s hemodynamic status, imaging findings, technical feasibility, and overall clinical condition rather than serum βhCG values alone.

Although maternal survival is excellent (98.3%), only one maternal death has been reported, in the earliest published case from 1936 [3], when most diagnostic methods were unavailable, and clinicians were unaware of the condition. Today, modern, accurate imaging and imaging-guided management strategies for minimally invasive or non-surgical options improve cure rates and survival. The PSP group includes young, healthy females who can compensate even for severe bleeding. Large multicenter studies could theoretically answer all the questions, but due to the exceptional rarity of PSP, this review of published cases will remain the most comprehensive source of knowledge.

From a clinical perspective, increased awareness of PSP may facilitate earlier diagnosis and timely referral, particularly in women presenting with left-sided abdominal pain, positive pregnancy tests, and an empty uterus. Future research should prioritize standardized reporting of individual cases and the establishment of international registries to strengthen the evidence base for diagnosis and management.

### Limitations

This study has several limitations. The exact timing of splenic rupture cannot be determined because many patients reported abdominal pain for several days before rupture. The nearly 90-year span of included cases further limits comparability, given substantial advances in diagnostic imaging, surgical techniques, perioperative care, and reporting standards. Post-intervention serum βhCG values were available in 13/60 reported cases (21.7%), and paired early relative decline could be calculated in only 12 cases, precluding meaningful analysis of postoperative hormonal dynamics. Earlier cases were often diagnosed intraoperatively or at autopsy, whereas contemporary cases benefit from advanced imaging modalities, such as CT and MRI. Similarly, treatment approaches have evolved from predominantly open surgery to minimally invasive or image-guided techniques for selected patients. These temporal changes may have influenced case detection, management strategies, and reported outcomes, limiting direct comparisons between early and contemporary cases. In addition, the absence of a control group restricts the ability to draw causal inferences.

Importantly, all included data are derived from case reports and small case series, which are inherently subject to publication bias, selective reporting, and a lack of standardized data collection. In addition, several variables were incompletely reported across cases, resulting in missing data and variable denominators for some analyses. The heterogeneity of reporting across studies further limits the comparability and synthesis of findings. Consequently, the observed frequencies and proportions should be interpreted with caution, as incomplete reporting may have influenced the estimated prevalence of individual clinical characteristics, diagnostic findings, and treatment approaches. This review was not prospectively registered, which may reduce methodological transparency compared with prospectively registered systematic reviews. Moreover, no formal risk-of-bias or methodological quality assessment was performed, limiting the ability to evaluate the reliability of the evidence. Therefore, the results of this review should be interpreted with caution. Also, publication bias is likely, as rare, unusual, diagnostically challenging, or successfully managed cases are more likely to be reported and published than uncomplicated or unpublished cases. Survivorship bias may also be present, as patients who survived long enough to undergo diagnosis and treatment are more likely to be reported in the literature. Consequently, the available evidence may not fully reflect the true clinical spectrum, severity, or outcomes of PSP.

## 5. Conclusions

PSP is extremely rare, and available case reports do not show a consistent pattern of potential risk factors, unlike the more common tubal ectopic pregnancy. Cases usually present between 4 and 14 weeks of gestation. Augustin’s triad, consisting of spontaneous initial pain in the left upper quadrant (sometimes flank/lumbar) pain, accompanied by amenorrhea of approximately two months, and vaginal bleeding/discharge, is present in half of cases. Ultrasound is the most common initial imaging modality, while CT and MRI frequently provide additional diagnostic information that may assist clinical decision-making in selected patients. Since 2010, MRI has become more common. Most implantations occur on the inferior and superior poles of the spleen. Surgical treatment is the predominant management approach, often involving laparotomy with splenectomy or partial splenectomy. Splenic rupture does not increase the rate of total splenectomy, although the number of cases is limited. Antibiotic prophylaxis is generally not documented, and therapeutic antibiotic use is reported in only two cases. No reliable βhCG threshold for medical management can be established based on the currently available evidence, and treatment decisions should be individualized according to hemodynamic stability, clinical presentation, and available expertise. Maternal survival is 98.3%.

## Figures and Tables

**Table 1:** Obstetric history in patients with primary splenic pregnancy (*n* = 60).

**Obstetric History**		n **(%)/Mean ± SD**
Parity	0	22 (36.7%)
	1	9 (15.0%)
	≥2	29 (48.3%)
Gravidity (number of previous pregnancies)	0	19 (31.7%)
	1	5 (8.3%)
	2	16 (26.7%)
	≥3	20 (33.3%)
Number of previous deliveries	0	23 (38.3%)
	1	9 (15.0%)
	2	23 (38.3%)
	≥3	5 (8.3%)
Previous spontaneous miscarriages/pregnancy losses	0	39 (65.0%)
	1	14 (23.3%)
	≥2	7 (11.7%)
Previous ectopic pregnancy	Yes	2 (3.3%)
	No	58 (96.7%)
**Maternal comorbidities**		n **(%)/Mean ± SD**
Comorbidities	None/not reported	56 (93.3%)
	Benign gynecological conditions *	1 (1.7%)
	Chronic medical conditions †	2 (3.3%)
	Other ‡	1 (1.7%)
**Fertility and contraception factors**		n **(%)/Mean ± SD**
Infertility/ovulation induction	Yes	3 (5.0%)
	No	57 (95.0%)
Intrauterine device (IUD)	Current/recent use	9 (15.0%)
	No	51 (85.0%)
Oral contraceptive use	Current/recent use	2 (3.3%)
	No	58 (96.7%)
**Previous abdominal/pelvic surgery**		n **(%)/Mean ± SD**
Previous abdominal/pelvic surgery	None	50 (83.3%)
	Non-gynecological (e.g., appendectomy, cholecystectomy)	4 (6.7%)
	Gynecological §	7 (11.7%)
Previous cesarean section	Yes	5 (8.3%)
	No	55 (91.7%)

* Benign gynecological conditions include, for example, benign ovarian cystic teratoid tumor. † Chronic medical conditions include long-term systemic diseases such as diabetes mellitus, asthma, hypothyroidism, or similar conditions; categories with multiple chronic conditions are grouped together. ‡ “Other” includes primary infertility, which was coded as a maternal comorbidity in the original dataset. § “Gynecological” surgery includes ovarian cystectomy, hysterectomy, myomectomy, tubectomy/salpingectomy, and/or cesarean section. Note that women who undergo both gynecological and non-gynecological procedures are counted in both categories, so the totals across rows may exceed 60.

**Table 2:** Clinical presentation and status at admission in women with primary splenic pregnancy (*n* = 60).

Characteristic	Category	Value, n (%)/Median (IQR; Range)
Pain characteristics		
Any pain	Present (any location)	52 (86.7%)
	No pain	8 (13.3%)
Pain location	Generalized/diffuse abdominal pain	11 (18.3%)
	Localized abdominal/flank pain	40 (66.7%)
	Chest pain	1 (1.7%)
	No pain	8 (13.3%)
Abdominal pain distribution	Generalized/diffuse abdominal pain	11 (18.3%)
	Left upper quadrant abdominal pain	29 (48.3%)
	Lower abdominal pain	5 (8.3%)
	Left-sided pelvic pain	4 (6.7%)
	Epigastric pain	1 (1.7%)
	Right upper quadrant pain	1 (1.7%)
Gynecologic symptoms		
Abnormal vaginal bleeding/discharge	Yes	25 (41.7%)
	No	35 (58.3%)
Other symptoms		
Amenorrhea	Yes	40 (66.7%)
Nausea	Yes	9 (15.0%)
Vomiting	Yes	9 (15.0%)
Dyspnea	Yes	1 (1.7%)
Tachypnea	Yes	3 (5.0%)
Diarrhea	Yes	1 (1.7%)
Constipation	Yes	0 (0.0%)
Anorexia	Yes	2 (3.3%)
Vital signs and physical examination		
Tachycardia	Yes	19 (31.7%)
Hypotension	Yes	25 (41.7%)
Fever > 38 °C	Yes	1 (1.7%)
Kehr’s sign	Present	12 (20.0%)
Abdominal tenderness	Present	32 (53.3%)
Guarding	Present	10 (16.7%)
Abdominal rigidity	Present	4 (6.7%)
Abdominal distension	Present	7 (11.7%)
Bowel sounds	Normal	1 (1.7%)
	Abnormal	0 (0.0%)
	Not audible	1 (1.7%)
	Unknown/not reported	58 (96.7%)
Anemia	Present	20 (33.3%)

**Table 3:** Diagnostic workup and imaging findings in women with primary splenic pregnancy (*n* = 60).

Characteristic	Category	Value, n (%)/Median (IQR; Range)	Concordant Findings, n/N (%)
Gestational age and pregnancy tests			
Gestational age at diagnosis, weeks	—	7 (IQR 6–9; range 4–14; *n* = 43)	—
Vaginal examination performed	Yes	24 (40.0)	NA *
	No	36 (60.0)	—
Cervical excitation test	Not done	55 (91.7)	—
	Negative	2 (3.3)	—
	Positive	3 (5.0)	—
Urine pregnancy test	Not done	28 (46.7)	—
	Negative	2 (3.3)	—
	Positive	30 (50.0)	—
Serum βhCG, mIU/mL	Preoperative/pre-intervention	21,002 (IQR 8544–36,996; range 46–711,431; *n* = 42)	—
	First postoperative/post-intervention measurement †	2900 (IQR 2000–5390; range 552–21,472; *n* = 13)	—
Imaging modalities			
Abdominal/Pelvic Ultrasound	Performed	35 (58.3)	25/35 (71.4)
	Not performed	25 (41.7)	—
Transvaginal Ultrasound	Performed	27 (45.0)	NA *
	Not performed	33 (55.0)	—
Abdominal/Pelvic CT	Performed	21 (35.0)	20/21 (95.2)
	Not performed	39 (65.0)	—
Chest CT	Performed	2 (3.3)	1/2 (50.0)
	Not performed	58 (96.7)	—
MRI of abdomen/pelvis	Performed	10 (16.7)	10/10 (100.0)
	Not performed	50 (83.3)	—
Chest X-Ray	Performed	0 (0.0)	—
	Not performed	60 (100.0)	—
Abdominal/Pelvic X-Ray	Performed	0 (0.0)	—
	Not performed	60 (100.0)	—
Preoperative differential diagnosis			
Preoperative differential diagnosis documented ‡	Yes	42 (70.0)	—
	Unknown/not reported	18 (30.0)	—

* Concordance was not assessed for transvaginal ultrasound and vaginal examination because these modalities were not intended to directly evaluate splenic pathology. † The first postoperative/post-intervention β-hCG corresponds to the first value recorded in the postoperative/post-intervention β-hCG column; the exact postoperative day was not consistently reported. ‡ Individual diagnostic categories were numerically coded in the original dataset, but the full codebook was not available; therefore, specific diagnostic labels were not reconstructed to avoid misclassification.

## Data Availability

The dataset generated and analyzed during this systematic review is provided in Supplementary Table S1. Additional extracted data supporting the summary tables are available from the corresponding author upon reasonable request.

## Supplementary Materials

The following supporting information can be downloaded at: https://www.mdpi.com/article/10.3390/jcm15156082/s1, PRISMA 2020 for Abstracts Checklist: Completed PRISMA 2020 for Abstracts checklist, PRISMA 2020 Checklist: Completed PRISMA 2020 checklist, Table S1: Published cases of primary splenic pregnancy with detailed data. This systematic review was reported in accordance with the PRISMA 2020 Statement.

## Abbreviations

The following abbreviations are used in this manuscript:

PSP	Primary Splenic Pregnancy
CS	Cesarean Section
CT	Computed Tomography
MRI	Magnetic Resonance Imaging
US	Ultrasound
βhCG	β human Chorionic Gonadotropin
MTX	Methotrexate

## Appendix A

The complete search strategy, including the full search strings and any filters applied for each database, was completed in July 2026. **PubMed** search included “splenic pregnancy” [Title/Abstract] OR “splenic ectopic pregnancy” [Title/Abstract] OR “spleen” [Title/Abstract] AND “pregnancy” [Title/Abstract] OR “spleen” [Title/Abstract] AND “ectopic pregnancy” [Title/Abstract]. **PubMed Central** search included “splenic pregnancy” OR “splenic ectopic pregnancy” OR “spleen” AND “pregnancy” OR “spleen” AND “ectopic pregnancy”. **Google Scholar** search included “splenic pregnancy” OR “splenic ectopic pregnancy” OR “spleen pregnancy” OR “ectopic pregnancy spleen”. **Scopus** search included TITLE-ABS-KEY (“splenic pregnancy” OR “splenic ectopic pregnancy” OR (“spleen” AND “pregnancy”) OR (“spleen” AND “ectopic pregnancy”)).
